# Adaptation and psychometric properties of the school engagement and contextual factors questionnaires for Covid-19 and post Covid-19 context

**DOI:** 10.1371/journal.pone.0272871

**Published:** 2022-09-08

**Authors:** Laura Lara, Mahia Saracostti, Ximena de-Toro

**Affiliations:** 1 Departmento de Psicología Evolutiva y de la Educación, Universidad de Sevilla, Sevilla, España; 2 Facultad de Ciencias Sociales y Humanidades, Carrera de Psicología, Universidad Autónoma de Chile, Talca, Chile; 3 Núcleo Científico Tecnológico en Ciencias Sociales y Humanidades, Universidad de la Frontera, Temuco, Chile; 4 Escuela de Trabajo Social, Universidad de Valparaíso, Valparaíso, Chile; 5 School of Social Work, Pontificia Universidad Católica de Chile, Santiago, Chile; Universite du Quebec a Montreal, CANADA

## Abstract

School engagement has been demonstrated to be a relevant aspect in promoting students’ successful trajectories, a commitment that in its turn is influenced by contextual factors (family, teachers, and peers). Having instruments to measure these constructs allows decisions to be made to improve student retention, especially relevant in the context of uncertainty caused by covid-19. The aim of the study was to adapt and analyze the psychometric properties of questionnaires used to measure school engagement and contextual factors in the context of the pandemic with elementary school students in Chile. After adaptation of the instruments, through expert evaluation and focus groups with students, they were administered to 579 students in seventh and eighth grade (mean age = 12.79, 52% were boys), and to 334 students in fifth and sixth grade (mean age = 11.35, 38% were boys) in Chile. Confirmatory factor analyses showed that the two versions of the school engagement measurement instrument had an adequate fit with the original model of three correlated factors, cognitive, affective, and behavioral commitment. Similarly, these two versions of the instrument measuring the contextual factors had a good fit with the original model of three correlated factors, family, teachers, and peers. In addition, both versions of both questionnaires presented appropriate levels of internal consistency.

## Introduction

### School engagement and related factors

School engagement is considered a key concept to promote the retention of children and adolescents in the school system. From this perspective, school dropout would be the final stage of a process of socio-educational exclusion that is not restricted to the willingness of the student but includes a series of contextual and relational factors—mainly family, teacher and peer support—as well as more classical structural variables such as the socio-economic conditions of families and students or the level of school socio-educational vulnerability [1–4]. School engagement is understood as the active participation of the student in the educational process, from home and/or school. Committed students consider learning to be meaningful and are motivated and committed to their learning and their future [5, 6]. In this way, school engagement constitutes a construct that unifies necessary concepts to reach academic success, like motivation and student autonomy, which can be considered necessary parts of school engagement but are not enough to realize the engagement alone [7]. School engagement can be seen as the process or mechanism through which learning outcomes unfold over time [8]. It describes the quality of a student’s productive participation at school. It is also a mediator connecting motivational beliefs (e.g., academic self-concept) and contextual factors (e.g., classroom climate) to learning outcomes. Student engagement can be viewed as the outward manifestation of motivation [8]. Motivational beliefs refer to the internal processes that mediate how and why students engage in school activity [9]. Students’ engagement is improved when they nurture and an intrinsic motivation to learn, and when the learning environment supports their psychological needs. Motivation context and engagement have a complex interaction that unfolds dynamically during schooling. Psychological needs drive students to participate in school activities while developing relationships and navigating both academic and social challenges [10]. Students who participate in school activities feel more capable academically, feel a stronger connection to the school and receive more positive interactions from peers and teachers [11].

Meanwhile, there is evidence that school engagement is a multidimensional construct that integrates an affective, a behavioral and a cognitive dimension [2–4, 12–16]. The affective dimension refers to the level of the student’s emotional response to the educational institution and their learning process, characterized by a feeling of involvement with the school and a consideration of it as a worthy place to be a part of. Affective engagement provides enthusiasm to participate and persevere with school activities. Students that are emotionally engaged become part of the school community and understand that school is significant in their lives, understanding school’s important role in teaching abilities necessary to succeed in later life. It improves attitudes about classmates and teachers and disposes students towards completing schoolwork. The behavioral dimension includes the student’s interactions and responses, within the classroom, the school and in extracurricular contexts. This aspect of SE is a spectrum from low behavioral engagement, universally expected involvement (daily attendance) to stronger engagement and optional involvement (e.g., participation in student government). Finally, the cognitive dimension is the conscious investment of energy to construct complex learning that goes beyond the minimum requirements. Building on the concept of psychological involvement in study it includes the awareness and willingness to put in the mental effort necessary to understand complicated ideas and learn challenging skills. It is the deliberate application of energy to comprehend and analyze, going further than the minimum requirements, it grants the student a willingness to overcome difficult educational challenges and invest in mastery of new skills. It involves putting into practice self-regulation strategies and work towards meeting tough goals.

On the other hand, as mentioned, school engagement is a variable which is highly influenced by contextual/relational factors, and if these are known, it is possible to intervene to improve levels of school engagement [17]. According to the literature, there are three main factors that can influence school engagement trajectories and that acquire greater relevance, with a view to sustaining the socio-educational process, particularly in the context of health crisis and its subsequent stages: 1) family support; 2) peer support; and 3) teacher support [17–20]. The first factor refers to students perceiving that they are supported by their families in the learning process and when they have problems, helping them with homework, talking about what is happening at school, whether it is telematic, face-to-face or blended learning, encouraging and motivating them to work well. The second factor has been defined as the students’ perception of the interpersonal relationships between classmates, the concern, trust, and support between peers. Finally, the third factor refers to students’ perception that they are supported and motivated by their teachers to learn and that their teachers help them when they have a problem [5, 21].

### Educational backgrounds and the pandemic: The role of school engagement

The recent COVID-19 pandemic, which involved the prolonged interruption of face-to-face classes in different countries, or the switch to a rather hybrid classroom system, exacerbated existing educational disparities and inequalities, with important consequences that could be further exacerbated when uncertainty remains about how the virus will behave and its impact on educational processes [22].

In terms of learning, data collected in 157 countries concluded that the pandemic could affect the global level of schooling and learning, effectively reducing students’ actual years of schooling [23]. Similarly, studies conducted in France, the Netherlands, Germany, and Sweden [24] reinforce the relationship between lower learning and virtual learning, which would be even more evident for students with higher socio-economic vulnerability and low educational attainment prior to the pandemic, emphasizing that the effects of the pandemic would be felt even in contexts where schools have remained open.

According to Şeren et al. [25] and Zhao et al. [22] there is an idea that education will have been transformed when life after the pandemic begins and/or that it must be reformed, even more in a context where normality is not easily achieved as it is not known how the virus will behave, and while face-to-face education remains important, alternative systems are still being considered for a pandemic and post-pandemic phase. In addition, from the experience of the pandemic, it is suggested an ideal model for seeking differentiation in the classroom to cater for the diversity of their students would be a combination of both online and face-to-face learning opportunities. In such a context, a requirement is for students to become more active in understanding and charting their own learning paths, i.e. to be more committed to their studies.

Similarly, Darling-Hammond et al. [26], Domina et al. [27], Dorn et al. [28] and Zhao et al. [12] emphasize the effectiveness of learning processes in healthcare emergency contexts will vary significantly according to factors such as access to and quality of remote education, family support and school engagement. Then, it is proposed to reconsider the role of attendance as a key to new learning models in a context of diverse classroom modalities (face-to-face, online and hybrid) where the student’s time spent in class becomes more important in terms of commitment, participation, and results, making it more important to measure school engagement than attendance.

It is worth mentioning that contextual factors linked to school engagement have also been impacted by the pandemic, where peer and/or teacher support is hindered by physical distance, and family support is affected by the high stress situation experienced at home. COVID 19’s impact on learning outcomes and schooling in Chile [23] concluded that students’ degree of commitment to the pedagogical process is fundamental to their ability to develop autonomous learning, and this commitment decreases with the expansion of the virtual modality due to the weakening of the student-school relationship.

### Measuring school engagement in pandemic times

Based on its relevance as a predictor of educational trajectories, a set of instruments measuring school engagement and contextual factors were designed and validated in the Chilean context for students in the upper primary and secondary school before the pandemic [14, 21, 29] which are the first instruments developed in Spanish to measure school engagement and which have also been validated for use with school adolescents in Colombia, Uruguay, Spain and Peru [30, 31]. There are no other instruments developed and/or validated for evaluating these constructs for the Chilean population.

Since school engagement and contextual factors measurement instruments were designed in a period characterized by daily school attendance, there was a need to adapt the instruments for a non-face-to-face or hybrid classroom context, but still be relevant in a face-to-face context, so that they can be relevant to apply them in a pandemic or post-pandemic context. Hence, the aim of this article is to adapt and analyze the psychometric properties of both questionnaires for the above-mentioned context.

## Material and methods

### Participants

The sample used for the analyses of the 7th and 8th grade version consisted of 579 students (n = 298 and n = 281, respectively) from municipal schools in two regions of Chile (Bernardo O’Higgins and Valparaíso). Ages ranged from 11 to 16 years (M = 12.79, SD = 0.89), 52% were boys (n = 301) and 48% girls (n = 278). The sample used for the analyses of the 5th and 6th grade version consisted of 334 students in 5th and 6th grade (n = 67 and n = 267, respectively) from municipal schools in three regions of Chile (Bernardo O’Higgins, Valparaíso, and Los Lagos). Ages ranged from 10 to 15 years (M = 11.35, SD = 0.78), 38% were boys (n = 127) and 62% girls (n = 207).

### Instruments

School Engagement Questionnaire (SCQ). It is a self-report instrument composed by 29 items that assesses three dimensions of school engagement: affective (10 items), cognitive (12 items) and behavioral (7 items), with a Likert response scale ranging from 1 (never or almost never) to 5 (always or almost always). In this article we use the developed version for final levels of elementary and middle school [14] and the version for initial levels of elementary school [29]. The questionnaire was initially developed with students from 7th grade to 1st secondary school, revealing favorable psychometric evidence for both its three-factor internal structure and internal consistency for the total scale (ordinal alpha of .95) as well as for three factors (.83 for affective, .86 for behavioral and .87 for cognitive commitment). Subsequently, a version was developed for students in 5th and 6th grade [29], with similar items but adapted to their developmental stage. The 5th and 6th grade version [29] has demonstrated the same factor structure and adequate levels of internal consistency both for the total scale (Omega index of .93) and for each of its component factors (.88 for affective, .85 for behavioral and .91 for cognitive commitment).

Contextual Factors Questionnaire (CFC). It is an instrument made up of 18 items that assess the three main contexts that influence school engagement: family (3 items), teachers (8 items) and school peers (7 items), with a Likert response scale ranging from 1 (never or almost never) to 5 (always or almost always). The questionnaire was initially developed for students in 7th grade to 1st secondary school [21], showing favorable psychometric evidence both for its three-factor internal structure and internal consistency for the total scale (ordinal alpha of .92) and for each of its component factors (.69 for the family factor, .83 for the teacher factor, and .83 for the peer factor). Subsequently, a version was developed for students in 5th and 6th grade [29], with similar items but adapted to the developmental stage of the target group, which showed the same factor structure and adequate levels of internal consistency not only for the total scale (Omega index of .91) but also for each of its component factors (.76 for the family factor, .88 for the teacher factor, and .89 for the peer factor).

Both versions of the two questionnaires were adapted to the Covid-19 context through three successive phases, in order to make them relevant to both face-to-face and distance learning. In the first phase, four members of the research team reviewed the questionnaires and made proposals for adapting the items to the Covid-19 teaching context, which were consolidated by a fifth member of the research team and submitted for expert review. In the second phase, seven teachers of 7th and 8th grade, and seven teachers of 5th and 6th grade reviewed the questionnaires (each group separately) with proposed changes made by the research team and made judgments on the adequacy of the items, suggesting, in case of inadequacy, possible alternatives for their adequate understanding by the targeted population. Teachers were contacted by email and analyzed the questionnaires individually using a document created for this purpose and sent to them via email. Once the responses were received, the suggested changes were analyzed. As a rule, items in which no expert pointed out any problems were kept as they were written, while those items in which all experts suggested a similar change were directly modified, finally, changes were provisionally proposed in those items in which more than one teacher pointed out problems, to be subsequently tested in the discussion groups by presenting both the original alternative and the one suggested by the teachers. In the third phase, four discussion groups (one per school year) were held with the target children and adolescents, where the appropriate understanding of the changes proposed in the two previous phases was analyzed. Each group was made up of six students (3 boys and 3 girls), they were conducted online, using the Zoom application, and took an average of 40 minutes. In the present study we include the main contents of the items based on the 7th and 8th grade version. Final versions of the Spanish questionnaires are available in the S1 Appendix. In this way, the 5th and 6th grade version is similar in the number and content of the items to the 7th and 8th grade version but has some differences in the writing and words (for example, the use of specific words more commonly used in 5th and 6th grades, grammatically simpler phrases in some cases or use more adequate examples).

### Analysis plan

The analysis of the factor structure of the questionnaires was carried out by means of Confirmatory Factor Analysis (CFA), with the aim of confirming whether the structure of the questionnaires was still appropriate to the COVID-19 context after the changes made in the adaptation process. The CFAs were conducted using Mplus 7.3, on the polychoric correlation matrix, with the Unweighted Robust Least Squares (ULSMV) estimation method. For each analysis we report chi-square statistics, Root Mean Square Error of Approximation (RMSEA) and associated 90% confidence interval (CI), Comparative Fit Index (CFI), and the Tucker-Lewis Index (TLI). For the CFA fit indices, the cut-off points established by Kline [32] were considered, including RMSEA < .08, CFI > .90, and TLI > .90. The use of multiple fit indices provides a more holistic view of goodness of fit [33].

Given the ordinal nature of the data, the internal consistency of the instruments was estimated using McDonald’s Omega coefficient, which is based on the matrix of polychoric correlations using the FACTOR program, both for the total number of scales and for each of their dimensions. In addition, Cronbach’s alpha was calculated, despite being based on Pearson’s correlation matrix, because it is the most widely used index in the social sciences [34], using the SPSS software.

### Procedure

Prior to data collection, permissions were obtained from the schools, informed consent signed by the students’ legal representatives as well as by the participating students. Due to the pandemic situation caused by Covid-19, at the time of data collection the students were away from the schools in which they were enrolled, so they completed the instruments remotely outside the school. This data collection was carried out collectively during class hours, through the virtual platform used by the students to attend their classes. Staff from the research team, duly trained for this purpose, provided the relevant explanation and were present throughout the filling in of the questionnaires to attend to and resolve any possible queries. The school engagement questionnaire was completed through a computerized platform developed for this purpose [5, 35, 36], with an approximate duration of between 30 and 40 minutes. This study received the approval of the Ethics Committee of the Universidad Autónoma de Chile (for the versions intended for seventh and eighth grade students) and of the Universidad de Valparaíso (for the versions intended for fifth and sixth grade students). The present study is the first step of a wider project that aims to develop and validate a system of strategies to promote school engagement (for more information, see [35]).

## Results

### Analysis of factor structure

Starting with the School Engagement Questionnaire, CFAs were conducted to test if the data fit the structure of a three-factor correlated model in each of the versions, whereby school engagement is composed of the three expected factors (affective, behavioral, and cognitive) in a correlated manner (three-factor correlated model). The results of the CFA for the version for 7th and 8th grade students show an acceptable fit of the data to the model (χ^2^ = 964.314; *df* = 374; RMSEA = .052/CI = .048- .056; CFI = .925; TLI = .919) as well as for the version for 5th and 6th grade students (χ^2^ = 621.912; *df* = 374; RMSEA = .045/CI = .038-.051; CFI = .930; TLI = .924).

The standardized factor saturations of the items in the belonging factors ranged between .462 and .934 for 7th and 8th grade version and between .311 and .841 for the 5th and 6th grade version (Table 1). In the version for 7th and 8th grade students, the correlation between the affective and cognitive factors was .744, between the affective and behavioral factors .361 and between the cognitive and behavioral factors .436. In 5th and 6th grade version, the correlation between the affective and cognitive factors was .759, between the affective and behavioral factors .364 and between the cognitive and behavioral factors .414.

**Table 1:** Items content and factor loadings of the school engagement questionnaire.

Factors and Items	F	
	7th & 8th	5th & 6th
*Affective*		
1. I feel like I am part of the school.	.597	.577
5. I can be myself at this school.	.621	.527
7. Most of the things I learn in school are useful.	.662	.699
8. Most teachers are concerned that the subject we learn is useful.	.689	.674
12. I am proud to be at this school.	.694	.728
15. What we do at school is very important to me.	.825	.841
19. They treat me with respect in this school (face to face or online).	.594	.581
22. What I learn in class is important to achieve my future goals.	.749	.761
27. I feel that the school cares about me.	.656	.575
29. I feel good at this school (face to face or online).	.712	.723
*Cognitive*		
2. Before an exam, I plan how to study the subject.	.583	.538
6. I use different resources (such as the internet or books) to search for supplementary information provided by the teacher.	.462	.612
10. When I am doing an activity, I make sure to understand everything possible.	.770	.636
13. After an exam, I wonder if my answers were correct.	.613	.639
14. I know what study strategies and habits I must change to improve and get better grades.	.673	.634
17. When I start an assignment, I think about the things I already know about the topic because that helps me understand better.	.689	.712
18. When I study, I write down new words, doubts, or important ideas.	.617	.635
20. For me it is important to understand the assignments and subjects well.	.758	.784
21. I know how to use different techniques and strategies to do my assignments (such as planning work, highlighting main ideas, discussing in groups, learning by phone or by computer, etc..	.712	.685
24. After finishing my assignments (or online assignments), I check if they are OK.	.717	.731
25. When I finish an assignment, I think about whether I have achieved the goal I had set for myself.	.748	.708
26. I pay attention to the comments that teachers make about my work.	.723	.790
*Behavioral*		
3. I skip classes, or I play hooky (or I do not connect to virtual classes).	.586	.725
4. I leave the classroom without asking permission (or I leave the online classes.	.573	.578
9. I am usually late for class (or I late for online classes).	.523	.311
11. Teachers have arranged to see my parents or guardians because of my bad behavior (or they have contacted my parents or guardians online.	.611	.499
16. I behave well in class (face to face or online).	.934	.792
23. I argue or fight with my classmates in the classroom (or during online classes.	.594	.610
28. They send me to the principal’s or counselor’s office because of my bad behavior (or the director or general inspector quotes me online).	.516	.960
**Total**		

*Note*: F = Factor loading

With regard to the Contextual Factors Questionnaire, CFAs were carried out to test if the data fit the structure of a three-factor correlational model for each of the versions: The contextual factors are composed of three factors representing family, teacher and peer support, which are correlated (Three-factor correlated model). The results of the CFA show a good fit of the data to the model for both 7th and 8th grade version (χ^2^ = 414.047; *df* = 132; RMSEA = .061/CI = .054-.067; CFI = .963; TLI = .957) and for the 5th and 6th grade version (χ^2^ = 293.889; *df* = 132; RMSEA = .061/CI = .051-.070; CFI = .969; TLI = .964).

The standardized factor saturations of the items in the belonging factors ranged between .670 and .865 for 7th and 8th grade version and between .562 and .882 for 5th and 6th grade version (Table 2). In the version for 7th and 8th grade students the correlation between the teachers and family factor was .721, between peers and family .545 and between teachers and peers .604, while the standardized factor saturations ranged between .670 and .865. In 5th and 6th grade version, the correlation between the family and teachers’ factor was .822, between family and peers .620 and between teachers and peers .700.

**Table 2:** Items content and factor loadings of the contextual factors questionnaire.

Factors and items	F	
	7th & 8th	5th & 6th
*Family*		
**1.** I talk to my family about what I do at school (or in online classes).	.722	.702
**2.** My parents or guardians motivate me to work well at school (or in online classes).	.829	.833
**3.** When I have a problem, I get help from my family.	.792	.815
*Teachers*		
**4.** My teachers want me to learn a lot.	.801	.845
**5.** When I have a problem, I get help from a teacher.	.779	.785
**6.** Teachers encourage me to do an assignment again if I make a mistake.	.708	.807
**7.** Teachers take an interest in me and help me if I have trouble doing an assignment.	.856	.882
**8.** I get along with my teachers.	.769	.817
**9.** Teachers care about me not only as a student but also as a person.	.818	.854
**10.** At the school, teachers and other adults treat all students with respect].	.722	.711
**11.** In this school, everyone’s participation and opinion are valued.	.701	.735
*Peers*		
**12.** My classmates support me and care about me.	.813	.769
**13.** I can trust my classmates.	.805	.793
**14.** My classmates are important to me.	.865	.862
**15.** I get along with my classmates.	.819	.881
**16.** I feel that I am important to my classmates.	.798	.783
**17.** At my school, at least one classmate supports me with difficult assignments.	.670	.562
**18.** When I do not understand something, my classmates help me to understand.	.776	.731
**Total**		

*Note*: F = Factor loading

### Reliability analyses

Reliability analyses showed that both the full scale and its component dimensions show adequate internal consistency in both questionnaires and for both versions (Table 3).

**Table 3:** Reliability of the questionnaires.

Questionnaire and Factors	α		ω	
	7^th^ and 8^th^	5^th^ and 6th	7^th^ and 8th	5^th^ and 6th
School Engagement (Total)	.907	.897	.906	.960
*Affective*	.859	.842	.896	.889
*Cognitive*	.885	.880	.910	.910
*Behavioral*	.718	.625	.818	.792
Contextual Factors (Total)	.914	.920	.966	.967
*Family*	.753	. 736	.825	.827
*Teachers*	.881	.895	.921	.936
*Peers*	.898	.880	.922	.912

Note: α = α Cronbach; ω = ω McDonald

## Discussion

From the results obtained, we can conclude that school engagement and contextual factors questionnaires in both face-to-face and distance learning contexts present adequate psychometric properties. Starting with the School engagement Questionnaire, the results of the factorial structure analysis showed that both the 7th and 8th grade version and the 5th and 6th grade version obtained a good fit of the data to the structure of a model of three correlated factors (affective, behavioral, and cognitive), which coincides with the theoretical configuration of the concept in pre-pandemic context [13, 14, 29, 30]. In addition, reliability analyses of the questionnaire showed an adequate internal consistency of the entire scale and its component dimensions. In relation to the Contextual Factors Questionnaire, the results of the factor structure analyses showed that both the 7th and 8th grade version and the 5th and 6th grade version obtained a good fit of the data to the structure of a three-factor model (family, teacher, and peers) as well as in the pre-pandemic context [15, 18, 29, 30, 37]. Meanwhile, reliability analyses showed that both the complete scale and its component dimensions show adequate internal consistency.

So, as a result, we have tools to measure school engagement and contextual factors that can be used by educational institutions to inform decision-making to promote positive educational trajectories based on evidence consistent with the current health situation. Since these instruments can be applicable in a remote classroom and/or hybrid classroom scenario, they are relevant in light of the uncertainty that the current health situation has generated around changing educational methodologies or in light of changes that schools themselves may wish to incorporate based on successful online experiences [22]. In both pre-pandemic and pandemic scenarios, measuring school engagement would be a more reliable indicator of educational processes and the degree to which students participate in educational activities, compared to attendance [38]. Thus, the absence of defined attendance measures during the pandemic challenges schools to find other measures to monitor educational trajectories, being school engagement a variable highly pertinent since it as it provides more complex information to inform decision-making within schools. The additional value of this study is that there are no other instruments that measure school engagement in a virtual context. Furthermore, it has been studied that school engagement is a prior variable and allows predicting the risk factors of dropout such as low performance, interrupted attendance for long periods, repetition, expulsion from the school, and finally, school dropout [15], whose figures increased sharply in Latin America, and could continue to increase due to the economic consequences that the pandemic has brought [39].

Certainly, the context of virtual classes brings new challenges, so it cannot be assumed that what happens in a face-to-face context can be replicated in a virtual context. For instance, items such as “I skip classes” lose pertinence, so participation should consider a context of virtual or hybrid classes, such as what is currently happening in Chile from the temporary closure of schools when cases increase. This is important because it seems that online schools, digital tools, and hybrid methodologies are here to stay, even after the pandemic [22]. On the other hand, contextual factors linked to school engagement have also been impacted by the pandemic, where peer and/or teacher support has been hampered by physical distance, as well as family support due to the high-stress situation experienced at home [40]. This situation is worrying given that lower student connection with peers, teachers and the wider school community is associated with higher dropout rates [19], which implies high social and individual costs [41–43]. Regarding this point, in the case of Chile, many teachers perceive less family support [44], to which is added a significant burnout within the teaching team. Hence the importance of having updated information in the context of online learning, so schools and districts can evaluate the effectiveness or impact on school engagement of the several initiatives and resources allocated to confront education in a pandemic context [45].

Similarly, Rivera [46] points out that online education tends to have higher dropout rates than face-to-face education, due to the fact that the former involves a higher autonomy level, which younger students would not have, in combination by the fact that students from more vulnerable sectors would have less family and economic support to join virtual classes.

Therefore, it is important to maintain as high levels of school engagement as possible, given its association with better academic results, greater interest in learning, greater social skills, student well-being and academic resilience [47–49]. Moreover, strong school engagement is an essential protective factor against social and educational problems, by preventing school dropout [13, 50] while promoting positive educational and social outcomes for students [51], such as reduced risk of substance uses and offending behaviors [52]. Likewise, it is a factor that minimizes learning loss and increases mental health, mitigating pandemic and post-pandemic stress.

Although there is limited research about school engagement in online settings, the research suggests considerations for how to promote engagement and support students’ self-regulatory practices. Some promising practices are to combine synchronous with asynchronous learning (e.g. video instruction, online activities) which provides more flexible and equitable opportunities for students to learn; privilege synchronous instances for wellness checks (e.g. morning meeting), small-group instruction, meetings with teachers, or spaces to share between peers; to improve access to internet and devices for online learning such as a laptop or tablet; to provide additional support for social and emotional learning; to prepare several contingency plans in accordance to public health policies. Additionally, school engagement and contextual factors may be influenced by different pedagogical practices which are able to be implemented in online setting [53].

On the other hand, according to the literature it is advisable that the measurement of school engagement and promotion strategies whose effectiveness has scientific evidence being part of a comprehensive system for monitoring school engagement and established within school policies to give it continuity regardless political changes [35]. An example is the System for the Evaluation, monitoring and follow-up of school engagement and contextual factors developed in Chile, available at: https://compromisoescuela.com/. This system proposes a six-step system for making decisions about intervention in school engagement and its contextual factors, based on scientific evidence. Some of the suggested interventions, which can be applied in a face-to-face or virtual context, are: communicate with families via e-mail, phone, postal mail, and social media to enhance family support; goal setting and formative feedback to promote cognitive engagement; online discussion spaces and peer collaborative learning strategies to promote peer support; or recognize the voice of students in decision-making to strengthen students’ feelings of belonging to their school or affective engagement.

Finally, the measurement of contextual factors and school engagement can open new discussions within schools in relation to traditional teaching practices. Interestingly some research evidence shows increased engagement among students during online and remote learning (based on for example the possibility of making a personal connection with their teachers by sharing more about their home life or interests via video conference) and pointed to the need to consider how online approaches to instruction could be beneficial to some students even during normal school operations [54].

However, it should not be ignored that education in times of pandemic is not just any virtual education but is immersed in a highly stressful context for students, teachers, and families [49]. Therefore, it is important to contextualize this type of measurement and its results, considering connectivity problems that affects thousands of families [55], mainly in rural areas, and how the pandemic has made the economic situation of many families more precarious. Previous gaps in the educational system in Latin America and the unequal conditions of access to human, economic, infrastructure and educational equipment and resources have been aggravated by the pandemic. Figures from UNESCO and the Inter-American Development Bank [56] show that learning was low at all educational levels, with greater severity in the case of students from more vulnerable families. Thus, an average percentage increase of 7% in school exclusion for economic reasons has been estimated for the Latin American and Caribbean region. Therefore, a limit of this study is that it focuses on those variables that are moldable by the school, however there are structural factors that have become more acute during the pandemic and that must be addressed hand in hand with school engagement to prevent a worsening of educational exclusion, such as families economic and health vulnerabilities.

## Supporting information

S1 Appendix (DOCX)

S1 Data (SAV)
