# Supplementary material for: Adaptation and psychometric properties of the school engagement and contextual factors questionnaires for Covid-19 and post Covid-19 context
Source: PLoS One. 2022 Sep 8;17(9):e0272871. doi: 10.1371/journal.pone.0272871 (PMC9455875; doi:10.1371/journal.pone.0272871)
Supplement: S1 Appendix — (DOCX) [file pone.0272871.s001.docx]

**Appendix**

**Final version of the School Engagement Questionnaire**

| *Nº* | 7^th^ and 8^th^ grade version | 5^th^ and 6^th^ grade version |
| --- | --- | --- |
| 1 | Siento que soy parte del colegio. | Siento que soy parte de esta escuela. |
| 2 | Antes de una prueba, planifico cómo estudiar la materia. | Antes de una prueba, reviso cómo estudiar la materia. |
| 3 | Me escapo de clases o hago la cimarra (o no me conecto a las clases virtuales). | Me escapo de clases o me corro de clases (o no me conecto a las clases en línea). |
| 4 | Salgo sin pedir permiso de la sala (o salgo de la clase virtual). | Salgo sin pedir permiso de la sala (o salgo de la clase en línea). |
| 5 | Puedo ser yo mismo(a) en este colegio. | Puedo ser yo mismo(a) en esta escuela (presencial o virtual). |
| 6 | Utilizo distintos recursos (como internet o libros) para buscar información complementaria entregada por el profesor. | Uso distintos recursos (como internet o libros) para entender mejor la información entregada por el profesor (a). |
| 7 | La mayoría de las cosas que aprendo en el colegio son útiles. | La mayoría de las cosas que aprendo en la escuela sirven para mi vida. |
| 8 | La mayoría de los profesores se preocupan de que la materia que aprendamos sea útil. | La mayoría de los profesores(as) se preocupan de que la materia que aprendamos sirva para mi vida. |
| 9 | Llego atrasado(a) a clases (o llego atrasado(a) a clases virtuales) | Llego atrasado(a) a clases (presenciales o en línea). |
| 10 | Cuando estoy haciendo alguna actividad, me preocupo de entender todo lo posible. | Cuando estoy haciendo alguna actividad, me preocupo de entender lo más posible. |
| 11 | Mis apoderados han sido citados por mi mala conducta (o han contactado a mis apoderados de manera virtual). | Mis apoderados han sido citados por mi mala conducta (en el contexto de las clases en línea, han contactado a mis apoderados por mi mala conducta). |
| 12 | Siento orgullo de estar en este colegio. | Siento orgullo de estar en esta escuela (presencial o virtual). |
| 13 | Después de una prueba pienso si las respuestas fueron las correctas. | Después de una prueba, reviso si las respuestas fueron las correctas. |
| 14 | Sé qué estrategias y hábitos de estudio tengo que cambiar para mejorar y obtener mejores calificaciones. | Sé cómo cambiar mi manera de estudiar para mejorar y obtener mejores notas. |
| 15 | Para mí es muy importante lo que hacemos en la escuela. | Para mí es muy importante lo que hacemos en la escuela (presencial o virtual). |
| 16 | Me porto bien en clases (presenciales o en virtuales). | Me porto bien en clases (presenciales o virtuales). |
| 17 | Cuando comienzo una tarea, pienso en las cosas que ya sé sobre el tema porque eso me ayuda a comprender mejor. | Cuando comienzo una tarea, recuerdo lo que he aprendido de la materia porque eso me ayuda a comprender mejor. |
| 18 | Cuando estudio, anoto palabras nuevas, dudas o ideas importantes. | Cuando estudio, anoto palabras nuevas, dudas o ideas importantes. |
| 19 | Me tratan con respeto en este colegio (presencial o virtual). | Me tratan con respeto en esta escuela (presencial o virtual). |
| 20 | Para mí es importante lograr entender bien las tareas y la materia. | Para mí es importante lograr entender bien las tareas y la materia. |
| 21 | Sé cómo utilizar diferentes técnicas y estrategias para realizar bien mis tareas (como, por ejemplo, planificar el trabajo, destacar ideas principales, discutir en grupos, aprendo por el teléfono o por el computador, etc.) | Sé cómo utilizar diferentes formas de estudio para realizar bien mis tareas (como, por ejemplo, planificar el trabajo, repasar la materia, estudiar en grupos, aprender desde el celular o el computador, etc.). |
| 22 | Lo que aprendo en clases es importante para conseguir mis metas futuras. | Lo que aprendo en clases es importante para lograr lo que quiero en el futuro. |
| 23 | Peleo con mis compañeros/as en la sala (o durante las clases en línea). | Peleo con mis compañeros en la sala (o durante las clases en línea). |
| 24 | Después de terminar mis tareas reviso si están bien. | Después de terminar mis tareas (o trabajos virtuales) reviso si están bien. |
| 25 | Cuando finalizo una tarea, pienso si he conseguido el objetivo que me había propuesto. | Cuando termino una tarea, pienso si la hice bien. |
| 26 | Pongo atención a los comentarios que los profesores hacen sobre mis trabajos. | Pongo atención a los comentarios que los profesores hacen sobre mis trabajos (presenciales o virtuales). |
| 27 | Siento que soy importante para el colegio. | Siento que soy importante para la escuela. |
| 28 | Me mandan a la oficina del director o del inspector general por mi mala conducta (o el director o inspector general me cita de forma virtual). | Me mandan a la oficina del director o del inspector general por mi mala conducta (en el contexto de clases en línea, el director o inspector general me ha contactado por mi mala conducta). |
| 29 | Me siento bien en este colegio (presencial o virtual). | Me siento bien en esta escuela (presencial o virtual). |

**Final version of the Contextual Factor Questionnaire**

| Nº | 7^th^ and 8^th^ grade version | 5^th^ and 6^th^ grade version |
| --- | --- | --- |
| 1 | Hablo con mi familia sobre lo que hago en la escuela (o en las clases virtuales). | Hablo con mi familia sobre lo que hago en la escuela (o en las clases virtuales). |
| 2 | Mis padres o apoderados me animan a trabajar bien en la escuela (o en las clases virtuales). | Mis padres o apoderados(as) me animan a trabajar bien en la escuela (o me apoyan en las clases virtuales). |
| 3 | Cuando tengo un problema, recibo ayuda de mi familia. | Cuando tengo un problema, recibo ayuda de mi familia. |
| 4 | Mis profesores(as) quieren que aprenda mucho. | Mis profesores(as) quieren que aprenda mucho. |
| 5 | Cuando tengo un problema, recibo ayuda de algún(a) profesor(a). | Cuando tengo un problema, recibo ayuda de algún(a) profesor(a). |
| 6 | Los profesores me alientan a realizar nuevamente una tarea si me equivoco. | Los profesores(as) me animan a realizar nuevamente una tarea si me equivoco. |
| 7 | Los profesores se interesan por mí y me ayudan si tengo dificultades para hacer las tareas. | Los profesores(as) se interesan por mí y me ayudan si tengo problemas para hacer las tareas. |
| 8 | Me llevo bien con mis profesores. | Me llevo bien con mis profesores(as). |
| 9 | Los profesores se preocupan de mí no sólo como estudiante sino también como persona**.** | Los profesores(as) se preocupan de mí no sólo como estudiante sino también como persona (o se preocupan por mí en la educación virtual). |
| 10 | En mi escuela, los profesores y otros adultos tratan a todos los estudiantes con respeto. | En mi escuela, los profesores(as) y otros(as) adultos(as) tratan a todos los estudiantes con respeto. |
| 11 | En este colegio, se valora la participación y la opinión de todos(as). | En esta escuela, se valora la participación y la opinión de todos(as). |
| 12 | Mis compañeros(as) me apoyan y se preocupan por mí. | Mis compañeros(as) me apoyan y se preocupan por mí. |
| 13 | Puedo confiar en mis compañeros(as). | Puedo confiar en mis compañeros(as). |
| 14 | Mis compañeros(as) del colegio son importantes para mí. | Mis compañeros(as) de la escuela son importantes para mí. |
| 15 | Me llevo bien con mis compañeros(as) de curso. | Me llevo bien con mis compañeros(as) de curso. |
| 16 | Siento que soy importante para mis compañeros(as) del colegio. | Siento que soy importante para mis compañeros(as) de la escuela. |
| 17 | En mi colegio, al menos un(a) compañeros(a) me apoya con las tareas difíciles. | En mi escuela, al menos un(a) compañeros(a) me apoya con las tareas difíciles. |
| 18 | Cuando no entiendo algo, mis compañeros me explican. | Cuando no entiendo algo, mis compañeros(as) me explican. |
